# Process of landfill leachate pretreatment using coagulation and hydrodynamic cavitation oxidation†

**DOI:** 10.1039/d3ra04259f

**Published:** 2023-11-02

**Authors:** Yina Qiao, Riya Jin, Jingshuai Gao, Kun Wang, Yu Jiang, Jian Xiong, MengYe Jia, Zengdi He, Jiaoqin Liu

**Affiliations:** a School of Environment and Safety Engineering, North University of China Shanxi Taiyuan 030051 P. R. China qiaoyina@nuc.edu.cn; b Key Laboratory of Biodiversity and Environment on the Qinghai-Tibetan Plateau, (Tibet University & Wuhan University), Ministry of Education, School of Ecology and Environment, Tibet University Tibet Lhasa 850000 P. R. China

## Abstract

Landfill leachate poses a threat to the environment and human health, and its complex composition made it difficult to treat. Among the methods for treating landfill leachate, the physicochemical combination method is considered to have significant effectiveness, low cost, and application potential. In this study, we propose a new method of coagulation and hydrodynamic cavitation/chlorine dioxide (HC/ClO_2_) for treating landfill leachate. The optimal conditions for coagulation and HC/ClO_2_ treatment were investigated experimentally. Under the optimal conditions for coagulation, the COD removal rate was 60.14%. Under the optimal HC/ClO_2_ treatment conditions, the COD removal rate was 58.82%. In the combined coagulation and HC/ClO_2_ process, the COD removal rate was 83.58%. Thus, the proposed method can significantly reduce the organic load before subsequent biological treatment processes, thereby reducing the operation cycles and cost of biological treatment.

## Abstract 2

A new method of coagulation and hydrodynamic cavitation/chlorine was proposed to treat landfill leachate, and the combination treatment can significantly reduce the organic load of landfill leachate and improve its biodegradability.

## Graphical abstract

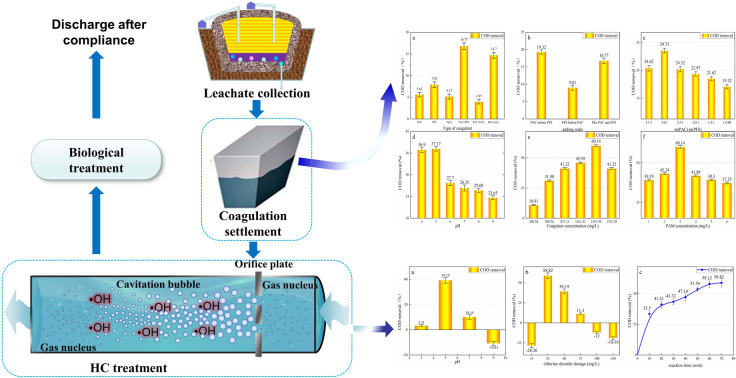

## 1 Introduction

Currently, there are three main ways to deal with domestic waste—sanitary landfill, composting, and incineration.^1^ The sanitary landfill method is the most widely used method because it offers the advantages of simple processing, convenient operation, relatively mature technology, low cost, and thorough disposal. However, landfill leachate, which is harmful to the environment, is produced in landfills. If the leachate is left untreated, it can pollute underground water sources and urban environments and affect human health.^2–4^ In view of this, the discharge standards for landfill leachate in various countries are becoming increasingly strict. To meet these standards, the effective treatment must be carried out before the discharge of landfill leachate.

It must be noted that landfill leachate has different reaction processes based on the age of the landfill. It leads to its composition varies significantly with the landfill's age. In general, Landfill leachate can be categorized into young, intermediate, and mature leachate.^5,6^ Young leachate is easy to treat biochemically, whereas intermediate and mature leachate are more difficult to treat owing to their complex composition—primarily, numerous organic pollutants, ammonia nitrogen, and heavy metals, as well as a high concentration of harmful compounds, such as aromatic and halogenated hydrocarbons.^7^ These toxic macromolecular organics directly contribute to the poor biodegradability of landfill leachate. Faced with the challenge of complex and difficult degradation of landfill leachate, it is crucial to explore an economical and efficient landfill leachate treatment process.

The characteristics of landfill leachate are generally expressed through basic parameters such as chemical oxygen demand (COD), biochemical oxygen demand (BOD_5_), BOD_5_/COD, pH, suspended matter (SS), ammonium nitrogen (N–NH_4_^+^), total Kjeldahl nitrogen (TKN), and heavy metals, among which COD and N–NH_4_^+^ are the main indicators used to measure the treatment effect of landfill leachate.^8,9^ The treatment procedure used for landfill leachate generally comprises pretreatment, biological treatment, and advanced treatment that primarily include physical, chemical, and biological treatment methods.^10,11^ Owing to the complex characteristics and high concentrations of ammonia nitrogen and heavy metals in landfill leachate, the effect of biological treatment is limited to some extent. Therefore, coagulation, advanced oxidation (AO) and other physicochemical treatment methods have received significant attention recently.

Coagulation is usually used for the pretreatment of the leachate. Some common coagulants and flocculants are alum, polyaluminum sulfate (PAS), polyaluminum chloride (PAC), polyferric sulfate (PFS), ferric chloride (FeCl_3_), and polyacrylamide (PAM). Coagulation–flocculation pretreatment can effectively remove COD, SS, chroma, and turbidity in leachate, and is particularly effective at removing refractory organic matter. Moreover, it is a low-cost method and has a stable effect. In practical applications, the water quality and quantity of leachate vary considerably, and the metal ion concentration of the effluent can increase if improper reagents are used. Therefore, the optimum reagent dosage should be determined through tests before application.

O_3_ and Fenton oxidation are widely used for leachate treatment in advanced oxidation processes (AOPs).^12^ There are two ways to use O_3_ to oxidize pollutants—under acidic conditions, O_3_ molecules directly attack pollutants electrophilically, whereas under alkaline conditions at a pH of 8–9, O_3_ decomposition produces ˙OH radicals, which act indirectly on pollutants.^13,14^ O_3_ oxidation offers the advantages of simplicity, mild conditions, fast degradation of organic matter, and no secondary pollution. However, O_3_ has strong selectivity, low solubility in water, a low utilization rate, and high costs, which limits its application in wastewater treatment.^15,16^ Fenton oxidation uses Fe^2+^ as a catalyst to oxidize and decompress organic matter in the leachate using ˙OH radicals that are generated through a reaction with H_2_O_2_.^17,18^ The Fenton process is simple, does not require a special reaction system, has a strong oxidation capacity and fast reaction rate, is less selective than O_3_, and its treatment effect is remarkable. However, the Fenton reaction process produces a large amount of sludge that requires secondary treatment, which increases costs and limits its large-scale application. Jegadeesan *et al.* used the aeration electrochemical Fenton process to treat landfill leachate, with optimal experimental parameters of a voltage of 4.5 V, H_2_O_2_ dosage of 5 g L^−1^, and pH of 3. Under these conditions, they achieved COD, BOD_5_, and color removal efficiencies of 99%, 95%, and 99%, respectively, after 90 min.^19^

In addition to O_3_ and Fenton oxidation, hydrodynamic cavitation (HC) is a special oxidation technology, which can speed up the oxidation process without the use of any additional additives.^20,21^ In this process, the fluid pressure changes owing to the limiting effect of the cavitation generator, leading to the birth, growth, and collapse of cavitation. When the cavitation collapses, local high temperatures (the hot-spot temperature in the bubble is 4700–5700 K and the temperature of the bubble wall is approximately 1900 K) and pressures (the pressure in the bubble exceeds 50 MPa) are generated, forming a strong shockwave and high-speed jet.^22,23^ The resulting energy is strong enough to destroy the chemical bonds of water molecules and the hydroxyl radical (˙OH, oxidation potential 2.8 eV) and strong oxidation occurs, acting on the pollutants and achieving degradation.^24^ Simultaneously, the extremely high temperature and pressure conditions generated during HC provide extreme chemical reaction conditions for pollutant degradation. HC can degrade organic pollutants solely by relying on the kinetic energy and pressure of water without additives or external energy. In addition, it is easy to operate and environmentally friendly. Therefore, HC has enormous application potential.

Some studies have found that HC combined with other AOPs, such as chlorine dioxide, can significantly improve the pollutant treatment efficiency.^25^ ClO_2_ is currently widely used as a disinfectant. Its oxidation potential is only 0.936 V. It is selective in the oxidation of organic matter.^26^ ClO_2_ alone has a poor treatment effect on pollutants. However, ClO_2_ can be combined with HC to develop and improve ClO_2_ wastewater treatment. Under the extreme conditions of HC, ClO_2_ will also produce secondary ˙OH radicals:^27^

1 ClO_2_ → ·O + ·ClO

2 ·O + H_2_O → 2·OH

3 3·O → O_3_

4 O_3_ + H_2_O → 2·OH + O_2_

Since HC combined with ClO_2_ (HC/ClO_2_) has a good synergistic effect on the removal of refractory organic matter, this method is theoretically feasible for the treatment of landfill leachate. The synergistic effect between HC and ClO_2_ is speculated to be due to the strong oxidizing action of ·OH and ·O produced during this process. The refractory organic compounds in landfill leachate such as polycyclic aromatic hydrocarbons may undergo displacement, addition, and dehydrogenation reactions, so that gradually open the ring to form chain organic compounds. Subsequently, large molecular chain organics are “torn” into smaller molecules during HC and are eventually completely oxidized and decomposed.^28–30^ In addition, the dissolution of ClO_2_ in the fluid can increase the gas content of the fluid and enhance cavitation.

Based on the above analysis, it can be considered to use the combined method of HC and ClO_2_ to treat aged landfill leachate. Aged landfill leachate has a low BOD_5_/COD, making it unsuitable for direct biological treatment. Therefore, physical and chemical treatment may be used before biological treatment, so as to effectively reduce the COD in landfill leachate and the toxicity of wastewater. Simultaneously, it can significantly reduce the burden of biological treatment, and improve the biodegradability of the leachate.^31^

This study aims to address the challenge of low BOD_5_/COD in the treatment of aged landfill leachate, and attempts to explore the impact of coagulation and HC/ClO_2_ methods on the pre-treatment of aged landfill leachate. It is expected to develop a low cost and low energy consumption treatment process for landfill leachate treatment to meet the economical and environment friendly requirements through this study.

## 2 Materials and methods

### 2.1 Materials

The wastewater treated in this study is leachate from aged garbage that was obtained from a municipal solid waste landfill in northern China. The water quality indicators are shown in Table 1. Raw water samples were stored at 4 °C, away from light and without pretreatment.

**Table 1:** Characteristics of landfill leachate

Parameter	Unit	Value
COD	mg L^−1^	2903.76
BOD_5_	mg L^−1^	319.41
BOD_5_/COD	—	0.11
pH	—	7.18
TOC	mg L^−1^	1489
N–NH_4_^+^	mg L^−1^	2063.14
SS	mg L^−1^	32
UV_254_	—	1.72

PFS, PAC, FeCl_3_, and PAM were selected as the complex. The coagulant components used in this experiment were polyaluminum chloride (PAC) and polyferric sulphate (PFS), and the ratio of the compound coagulant was m(PAC) : m(PFS) = 3 : 1.

The rest reagents were analytically pure. Sulfuric acid and sodium hydroxide were used to adjust the pH of the solution.

### 2.2 Experimental set-up

The experimental equipment for the coagulation process was a six-link electric agitator.

**Fig. 1:**
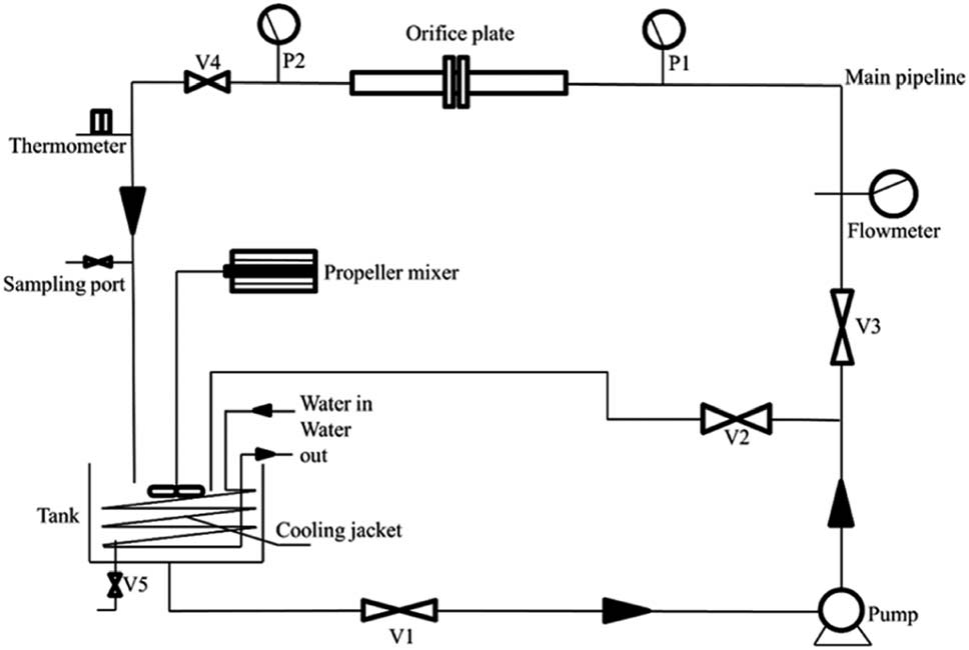
Schematic diagram of the flow in the HC system.

The experimental equipment for the HC process is a closed hydrodynamic circulation system with the HC generator (orifice cavitation) as its core. The equipment primarily comprises the HC generator, a high-pressure pump, self-priming pump, water tank, flowmeter, pressure gauge, pipe fittings, valves, *etc.*Fig. 1 shows the schematic of the flow in the system, and the detailed parameters and functions of the system are shown in Table S1.†

### 2.3 Analytical test methods

#### 2.3.1 Test methods

The BOD_5_ was determined through the dilution inoculation method stipulated in the Chinese national standard (GB/T 7488-1987). The COD was determined using the potassium dichromate method stipulated in the Chinese National Standard (GB/T 11914-1989). The COD was calculated according to the method described in text S1.†

#### 2.3.2 Dimensionless cavitation number

The cavitation number (*σ*_inlet_) is a dimensionless number that characterizes the cavitation state and inception; it is calculated as follows:^32^5
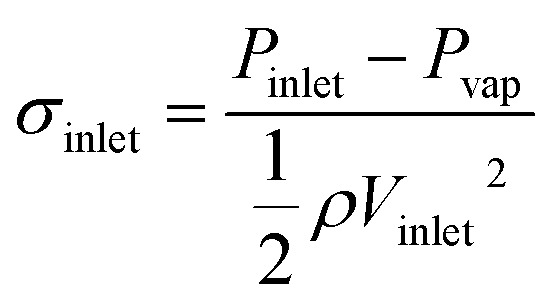
where *P*_vap_ was the vapor pressure; *P*_inlet_ and *V*_inlet_ were the pressure and velocity respectively at the reference section upstream of the venturi. The lower the value of *V*_inlet_, the more the cavitation develops, which implies that the contained gas volume in the cavitation basin is higher. As the inlet pressure increases, the cavitation number gradually increases, and the energy released in the collapse zone is higher.

### 2.4 Experimental procedure

During the coagulation stage, the mixing speed was set at 200 r min^−1^ and 50 r min^−1^ respectively for 2 min and 20 min respectively after each dosage of the reagent. Stir and stand was for 30 min. Take samples at 2 cm below the liquid level to detect the COD value and calculate the COD removal rate.

After determining the best coagulant addition sequence and the best compound combination, the influence of pH, coagulant dosage, coagulant aid dosage, and standing time on the treatment effect was investigated by measuring the change in the COD before and after the reaction.

The HC and ClO_2_ oxidation processes were evaluated individually. Annular orifice plates with different opening rates were used for the HC process to treat the landfill leachate (raw water). The treatment effect of ClO_2_ oxidation on the leachate (water after coagulation) was investigated considering different ClO_2_ concentrations, pH values, and reaction times.

The treatment effect of different values of pH, ClO_2_ dosage, and reaction time on COD in the leachate was investigated using the combined HC/ClO_2_ chemical oxidation treatment method. HC experiments were performed using the set-up shown in Fig. 1. The landfill leachate (50 L) to be treated was added to the HC tank, and the initial pH was adjusted to the required value using H_2_SO_4_ and NaOH. ClO_2_ was dribbled in less than a minute before the experiment began. After starting the HC system, the valve was rotated to adjust the inlet and outlet pressures to 4 and 0 bar, respectively. The COD of the wastewater was tested periodically, and each experiment was repeated thrice.

## 3 Results and discussion

### 3.1 Coagulation and complex

**Fig. 2:**
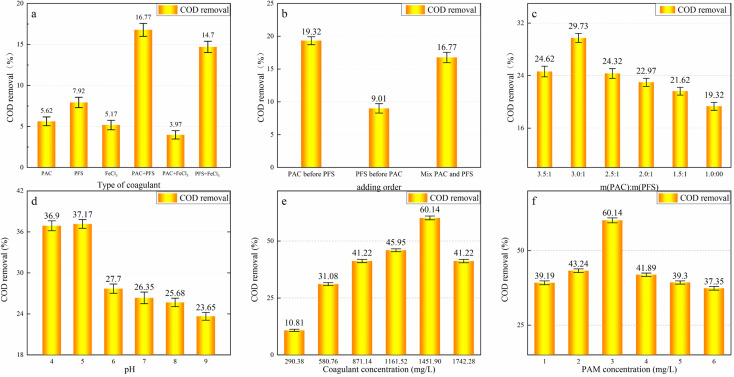
Study of optimal conditions for coagulation: (a) type of coagulant; (b) dosage sequence; (c) ratio of coagulant mixture; (d) pH; (e) coagulant dosage; (f) flocculant dosage. The concentration of coagulants in (a) was 600 mg L^−1^. PAC and PFS are used as the composition of the compound coagulant in (d) and (e). The ratio of PAC to PFS is m(PAC) : m(PFS) = 3 : 1.

The results of the experiments to determine the optimum conditions for coagulation are shown in Fig. 2. The initial reaction conditions were 1 g L^−1^ of coagulant mixed with a mass ratio of 1 : 1 and 3 mg L^−1^ of PAM solution. Each subsequent step was used to determine the best conditions for the coagulation process.

As shown in Fig. 2a, the hydrolysate of iron salt has a larger surface area than that of aluminum brine, making it more conducive to the adsorption of organic matter and suspended particles.^33,34^ As PFS can promote the precipitation of PAC flocs, the combination of PAC and PFS has the best COD removal effect. As shown in Fig. 2b, when PAC is added before PFS, the PAC has enough time to hydrolyze, and the flocculation generated by the subsequent addition of PFS can make up for the fine flocculation and poor sedimentation of PAC. Their combined action provides optimal COD removal. As shown in Fig. 2c, a 3 : 1 ratio of the m(PAC) : m(PFS) compound coagulant achieves the best treatment effect.

Fig. 2d indicates that COD removal changes slightly with a pH value of 4 and 5. The maximum COD removal of 37.17% occurred at a pH value of 5, and the COD removal rate gradually decreased when pH > 5. This is because the stability of the colloid is maintained by the interaction force between the colloids that keeps the particles suspended in the solution. The pollutants cannot condense and settle unless the pH value of the solution is adjusted to the equipotential point.^35^ When the pH value is low, the organic colloids adsorbed around the PAC + PFS hydrolysates reduce significantly as they combine with H^+^ in the solution to form organic acids, resulting in poor COD removal by coagulation. As the pH value increases, the hydrolyzed products of PAC and PFS are the cations from the single ·OH complex, multiple ·OH complex, polymers, hydroxide precipitate, *etc.* The cations produced by PAC hydrolysis can compress the double electron layer and reduce the ζ potential of the colloidal particles, further accelerating their destabilization.^36,37^ These multi-nuclear complex ions also have significant adsorption-bridging and precipitation net-capturing effects, and the strong adsorption of colloidal particles in the water adsorbs with the generated aluminum salt floc, thereby promoting floc sedimentation. When the pH value exceeds 6, the polynuclear complex ions that play a major role in the hydrolytic products of the coagulant reduce significantly, and the hydrolytic products are mostly converted into hydroxides. Consequently, the coagulation effect is substantially weaker, and the COD removal rate reduces. Based on an analysis of the experimental results, a pH value of 5 provides the best coagulation conditions.

The coagulant mixture ratio also affects COD removal. When the coagulant is added to the wastewater, the positively charged cationic and colloidal particles undergo a compression double layer, charge neutralization, and an adsorption bridging reaction, forming flocs and reducing the COD. However, when excess coagulant is added, charge repulsion occurs owing to the existence of excessive positively charged ions. Consequently, the sedimentation of the colloidal particles becomes weak, and the coagulation effect is worse. As shown in Fig. 2e, the best COD removal effect was obtained with m(coagulant):m(COD) = 0.6 (1451.80 mg L^−1^ of coagulant). Therefore, the optimal dosage of the coagulant is m(coagulant):m(COD) = 0.6.

PAM primarily causes colloidal settlement through adsorption bridging. When an appropriate quantity of PAM is added, the flocculant can adsorb small colloids and form larger flocs, which is conducive to settlement. When excess PAM is added, the surface of the colloidal particles is completely covered by the polymer flocculant. At this time, the charge of the colloidal particles is consistent with that of the flocculant polymer. The similarly charged particles repel each other and cannot condense, resulting in a poor COD removal effect. As shown in Fig. 2f, 3 mg L^−1^ of PAM is optimal.

In summary, PAC + PFS was determined to be the best coagulant, and the optimal reaction conditions are: pH = 5, m(PAC) : m(PFS) = 3 : 1, total dosage of PAC + PFS = 1451.80 mg L^−1^, and PAM dosage = 3 mg L^−1^. Under these conditions, the COD removal rate is 60.14%. The quality index of the coagulated effluent is shown in Table 2.

**Table 2:** Coagulant effluent index

	COD/(mg L^−1^)	pH
Water after coagulation	1157.58	4.8

### 3.2 Optimization of experimental conditions

#### 3.2.1 The treatment of landfill leachate with HC

**Fig. 3:**
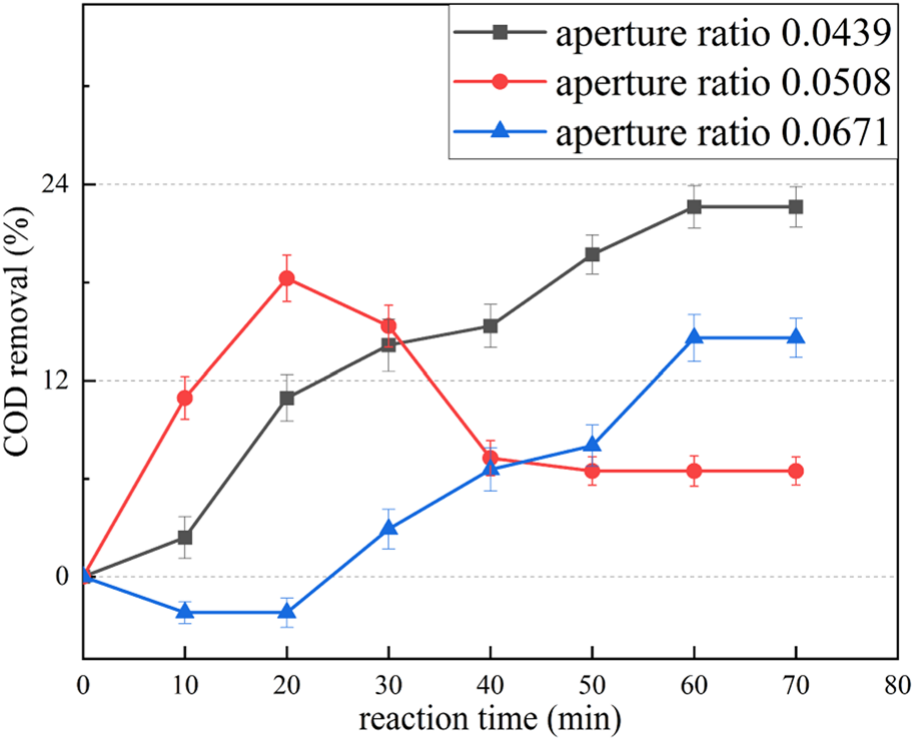
Leachate treated using only HC with different orifice opening rates.

Annular orifice plates with different opening rates were used to treat the landfill leachate using HC. The water sample index of the leachate is shown in Table 1. The results are shown in Fig. 3 and Table 3.

**Table 3:** Results of optimal orifice plate for leachate treatment, considering a reaction time of 60 min (orifice opening rate = 0.0439)

	COD	BOD_5_	BOD_5_/COD
Before HC treatment (mg L^−1^)	2903.76	319	0.11
After HC treatment (mg L^−1^)	2246.64	386	0.17
Rate of change	Decreased by 22.63%	Increased by 21.00%	Increased by 54.55%

With an orifice opening rate of 0.0439, the COD continues to decrease as the reaction time increases, decreasing to the lowest level after 60 min. With an orifice opening rate of 0.0508, the COD decreases rapidly during the first 20 min, rises sharply during 20–40 min, and remains unchanged thereafter. With an orifice opening rate of 0.0671, the COD first increases slightly above the initial value, then decreases slowly, and eventually becomes stable after 60 min. According to the principle of COD determination using potassium dichromate, acid potassium dichromate can completely oxidize straight-chain aliphatic compounds. However, some organic compounds, such as aromatic compounds and pyridine, have a stable structure and are difficult to oxidize, whereas some volatile organic compounds are difficult to fully contact with the oxidant solution owing to their presence in the gas equivalent of the reaction system. Consequently, the oxidation is not obvious. The increase in COD observed during the experiment may be attributed to the fact that the undetected COD in the raw water becomes detectable after HC treatment. Specifically, after the HC treatment, the macromolecular organic matter in the raw water transforms into small molecules and aromatic compounds like polycyclic aromatic hydrocarbons transform into chain organic matter that is oxidized by the acid potassium dichromate. This results in an increase in the COD. As the reaction time increases, the small organic matter molecules generated by the preceding reaction are further oxidized, resulting in a decrease in the COD (for example, at an orifice opening rate of 0.0671). During the reaction, the decomposition of the refractory organic matter and the mineralization of the easily degradable organic matter occur simultaneously. If the mineralization exceeds the decomposition, the COD decreases; if the decomposition exceeds the mineralization, the COD increases. At an orifice opening rate of 0.0439, the COD continues to decrease, proving that the percent of COD removal increases.

#### 3.2.2 The treatment of landfill leachate with ClO_2_

**Fig. 4:**
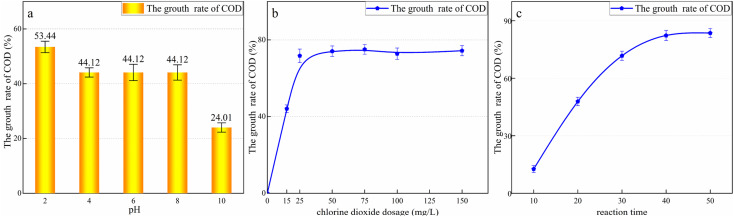
Effects of different factors on the treatment of leachate using ClO_2_: (a) pH; (b) ClO_2_ dosage; and (c) reaction time. The experimental conditions are as follows: (a) ClO_2_ dosage was 100 mg L^−1^, the reaction time was 40 min; (b) pH = 5, the reaction time was 40 min; (c) pH = 5, ClO_2_ dosage was 100 mg L^−1^.

Next, the landfill leachate was treated using ClO_2_ alone. The experimental sample was the effluent obtained after coagulation. The water quality index of the sample is shown in Table 2. The influence of the pH (Fig. 4a), ClO_2_ dosage (Fig. 4b), and reaction time (Fig. 4c) on the COD in the leachate was investigated using the control variable method.

To determine the effect of pH, the pH value of the wastewater was adjusted to 2, 4, 6, 8, and 10 using dilute H_2_SO_4_ or NaOH solution. A uniform dosage of 100 mg L^−1^ of ClO_2_ was added to the wastewater, and the samples were placed in the agitator and left to react for 30 min. The reaction was terminated by removing the excess ClO_2_ using 0.1 mol L^−1^ sodium thiosulfate. As shown in Fig. 4a, the COD of all the samples increased. This may be because the oxidation potential of ClO_2_ is only 1.51 V, which is much lower than that of ·OH (2.08 V). Considering the difficult to degrade organic molecules, although ClO_2_ cannot completely mineralize them, it destroys their molecular structure and forms secondary chain hydrocarbon molecules. Previous studies have found that ClO_2_ can only break up molecules and reduce the content of aromatic compounds.^38^ ClO_2_ can destroy the structure of organic molecules in the leachate,^39^ oxidizing macromolecules such as polycyclic aromatic hydrocarbons and humus into small molecules that are more easily oxidized by potassium dichromate. Therefore, the COD increases with the addition of ClO_2_.

At a pH value of 2, the COD increased by 53.44%. At a pH value of 4–8, the COD change rate stabilized, reaching 44.12%. Finally, at a pH value of 10, the COD increase was minimal at 24.01%. Thus, the oxidation potential of ClO_2_ is significantly affected by pH. The more acidic the solution, the stronger the oxidation capacity of ClO_2_, and the highest oxidation activity can be achieved under acidic conditions. Based on the experimental results, the subsequent ClO_2_ oxidation experiments were conducted at a pH value of 4.8 to avoid wasting the reagent and simplify the procedure.

As shown in Fig. 4b, the COD increased with the increase in ClO_2_ dosage. With a ClO_2_ dosage of 50 mg L^−1^, the COD peaked, increasing by 72.78%. A further increase in ClO_2_ dosage did not change the COD significantly. This is because the addition of excess ClO_2_ cannot further decompose the oxidation products.

To determine the effect of the reaction time, 500 mL of the coagulated effluent was placed in a beaker, 50 mg L^−1^ ClO_2_ was added, and the solution was stirred, with the COD value measured every 10 min. Before each measurement, the residual ClO_2_ in the sample was removed using sodium thiosulfate. The results are shown in Fig. 4c. As shown, the COD continued to increase with the increase in the reaction time up to 40 min from the beginning of the reaction. Subsequently, it remained almost unchanged, and the final COD increase rate was 82.31%. Thus, the optimal ClO_2_ dosage for treating landfill leachate is 50 mg L^−1^ and the optimal reaction time is 40 min.

### 3.3 Treatment of landfill leachate with HC/ClO_2_ after coagulation

After coagulation, the effluent was treated using HC/ClO_2_ chemical oxidation. The effect of pH, ClO_2_ dosage, and reaction time on the treatment of COD in the leachate was investigated.

**Fig. 5:**
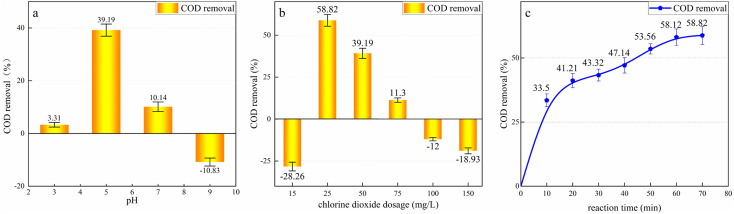
Influence of various factors on HC/ClO_2_ oxidation treatment: (a) pH; (b) ClO_2_ dosage; (c) reaction time. The experimental conditions are as follows: (a) ClO_2_ dosage was 50 mg L^−1^, the reaction time was 80 min; (b) pH = 5, the reaction time was 80 min; (c) pH = 5, ClO_2_ dosage was 50 mg L^−1^.

To determine the effect of pH on the treatment process, 50 L of the coagulated leachate was combined with 50 mg L^−1^ of ClO_2_ and four experiments were carried out at pH values of 3, 5, 7, and 9, with a reaction time of 80 min. The COD removal rate is shown in Fig. 5a. Under the optimal pH condition, different ClO_2_ dosages—15 mg L^−1^, 25 mg L^−1^, 50 mg L^−1^, 75 mg L^−1^, 100 mg L^−1^, and 150 mg L^−1^—were used to determine the optimal ClO_2_ dosage. The corresponding COD removal rates are shown in Fig. 5b. As shown in Fig. 5a, the treatment effect under neutral and acidic conditions was better than that under alkaline conditions. The highest COD removal rate of 39.19% was obtained at the pH value of 5. In contrast, the COD increased by 18.83% when pH > 7. This is because under alkaline conditions, CO_2_ generated by organic mineralization exists owing to HCO_3_^−^ and CO_3_^2−^, which are cleared by reaction with·OH:^40^

6 ·OH + CO_3_^2−^ → ·OH^−^ + ·CO_3_^−^

7 ·OH + HCO_3_^−^ → ·OH^−^ + ·HCO_3_

When pH = 9, disproportionation occurs and the oxidation capacity of ClO_2_ weakens. Approximately 10% of the total ·OH generated by the HC system diffuses into the liquid medium, and the rest recombines to form H_2_O_2_. Consequently, the concentration of ·OH in the liquid is low, resulting in less mineralization of the organic matter.^41^ At this time, the COD primarily increases owing to the physical action of cavitation.^42^

Acidic conditions are more conducive to improving ·OH utilization in the HC/ClO_2_ system, which is the primary reason for COD reduction. ClO_2_ has a higher oxidation potential under acidic conditions, and the CO_2_ generated by mineralization flows out of water as a gas instead of forming HCO_3_^−^ and CO_3_^2−^ and clearing ·OH. Under acidic conditions, the ·OH concentration at the cavity interface is higher, and the organic molecules are directly attacked by ·OH, significantly improving the ·OH utilization rate. It should be noted that a lower H^+^ concentration is not more beneficial for COD removal; however, the best COD removal rate was observed at pH = 5. This is because, under strong acidic conditions, a large amount of H^+^ combines with ·OH, consuming a large amount of ·OH and limiting the COD removal rate. Therefore, the pH value of 5 is the best condition to balance ·OH formation with the existence of organic matter,^43^ and the COD removal rate is most favorable at this condition.

Fig. 5b indicates that there is an optimal ClO_2_ dosage range. When the ClO_2_ dosage was 25 mg L^−1^, 50 mg L^−1^, and 75 mg L^−1^, the COD in the HC/ClO_2_ system decreased by 58.82%, 39.19%, and 11.3%, respectively. Outside this range, the COD increased.

During leachate treatment, the mineralization and decomposition of organic matter occur simultaneously. When the amount of mineralization exceeds that of decomposition, the COD decreases; when the amount of mineralization is less than that of decomposition, the COD increases. There are three oxidation modes in the HC/ClO_2_ reaction system—HC oxidation, ClO_2_ oxidation, and HC/ClO_2_ co-oxidation. COD reduction is primarily caused by the presence of free radicals in the HC/ClO_2_ system. When the ClO_2_ dosage is insufficient, the number of free radicals available is low. Consequently, the mineralization of organic matter is less than the decomposition of organic matter, and the COD increases. When excessive ClO_2_ is added, only a part of the added ClO_2_ is activated by HC to produce free radicals, and most of it reacts directly with the organic pollutants in the leachate. In this case, the oxidation of ClO_2_ is dominant in the HC/ClO_2_ system. ClO_2_ can only decompose organic matter and does not realize mineralization, leading to an increase in COD. When the ClO_2_ dosage is 25 mg L^−1^, the ClO_2_ is activated to the maximum extent, significantly improving the free radical conversion efficiency in the HC/ClO_2_ system. The amount of organic mineralization is far greater than that of organic decomposition, thereby realizing the maximum COD removal.

The influence of the reaction time on HC/ClO_2_ oxidation treatment is shown in Fig. 5c. During the first 20 min of the reaction, the COD decreased rapidly; after 20 min, the COD removal rate gradually decreased with the increase in the reaction time, and the final COD removal rate was 58.82%. The initial response of the reaction was swift. As the reaction progressed, ClO_2_ was gradually consumed, resulting in a slow reaction. After 60–70 min, the reaction almost stopped and the change in the COD was insignificant. Notably, the COD did not increase during the entire process, indicating that mineralization is always higher than decomposition during the reaction. The optimal reaction time is 70 min.

The above results indicate that HC/ClO_2_ can significantly improve the removal efficiency of pollutants from leachate. Under the optimal operating conditions, the COD removal rate is 58.82% and the COD remaining in the leachate is 476.69 mg L^−1^.

### 3.4 Mechanism

In our previous study,^44^ HC was used in combination with other AOPs (K_2_FeO_4_) to treat landfill leachate. A Fourier-transform near-infrared spectrometer (FT-IR) was used to detect and analyze the freeze-dried samples of the stock and treated leachate solutions.

The results revealed that the band intensity of the leachate after HC is significantly lower than that before HC. The absorption peak of the leachate treated with HC/K_2_FeO_4_ was primarily attributed to inorganic salt ions, which can be considered as evidence of a decrease in the number of organic compounds with these functional groups. Therefore, HC-enhanced AOPs can significantly promote the degradation of organic pollutants in landfill leachate.

As discussed in Section 3.3, the introduction of ClO_2_ into the HC system could theoretically generate more ·OH radicals to degrade pollutants. This was qualitatively verified using the fluorescence probe method in our previous experimental study.^45^ Therefore, the large number of ·OH radicals produced in an HC/ClO_2_ system significantly promote the degradation of organic pollutants in landfill leachate.

### 3.5 Energy requirement and cost analysis for the proposed treatment system

In the combined process, the pH conditions of the effluent after coagulation and the wastewater treated by cavitation are very close. Consequently, the pH value need not be adjusted again during actual operations, thereby saving both labor and material costs and simplifying the process. In practical application, the required amount of acid and base is small and can be replaced by waste acid and base. Therefore, the cost of the acid and base is not listed in Table 4.

**Table 4:** Reagent cost accounting

Reagent names	Unit price	Dosage of 1 m^3^ leachate	Cost (¥ per m^3^)
PAC	800 ¥ per t	1.089 kg	0.871
PFS	1700 ¥ per t	0.363 kg	0.617
PAM	8000 ¥ per t	3 g	0.024
ClO_2_	0.04 ¥ per 135 mg (approx.)	25 g	7.41 (approx.)
Total	—	—	8.92 (approx.)

The electricity cost primarily includes the electricity required for coagulation and HC. Compared with HC, the energy required for coagulation is negligible. Considering China as an example, the unit price of industrial electricity is 0.5 ¥ per kW h.

The electricity cost for 1 m^3^ leachate = 1000 L/50 L × 2.2 kW × 7/6 h × 0.5 ¥ per kW h = ¥25.67. The total cost = ¥8.92 + ¥25.67 = ¥34.59.

The instruments used in this experiment are at a laboratory scale, and sufficient power is selected for the water pump to meet the requirements of the experimental investigation. In actual wastewater treatment, a low power adaptive water pump can be used to reduce energy consumption, multi-pump operation can be employed to improve the treatment efficiency, and the distance between the HC and the pump can be shortened as far as possible to reduce the head loss. These measures can significantly improve electricity efficiency and reduce the cost of electricity. The above cost analysis is intended to serve only as a reference.

## 4 Conclusions

Sanitary landfills are the most commonly used method for treating solid municipal waste. However, the leachate produced from landfills is extremely harmful to the environment and the human body and must be treated before discharge. This study proposed an economical and environmentally friendly method to use coagulation and HC/ClO_2_ oxidation as pre-treatment processes which can significantly degrade organic pollutants in aged landfill leachate. The following conclusions were obtained.

For coagulation treatment, the optimal coagulant compound combination was PAC + PFS (PAC added before PFS), the optimal ratio of the compound coagulant was m(PAC) : m(PFS) = 3 : 1, the optimal dosage of the compound coagulant was 1451.80 mg L^−1^, the optimal dosage of PAM was 3 mg L^−1^, and the optimal pH value for the reaction was 5. Under these conditions, the COD removal rate was 60.14%.

For HC/ClO_2_ treatment, the COD removal rate was 58.82% after 70 min, with a pH value of 5, an initial ClO_2_ concentration of 25 mg L^−1^, and an inlet pressure of 0.4 MPa. The combined process of coagulation and HC/ClO_2_ can effectively treat landfill leachate, achieving a final effluent COD removal rate of 83.58%.

The HC experiment revealed that the optimal orifice opening rate is 0.0439. When treating leachate with ClO_2_ alone, the COD increases significantly, as does BOD_5_/COD. Although ClO_2_ does not reduce the COD concentration, it improves water quality and reduces biological toxicity. The combined HC/ClO_2_ method can play a key role in the degradation of organic pollutants in landfill leachate as it produces a large quantity of ·OH free radicals.

## Conflicts of interest

The authors declare no conflict of interest.

## Supplementary Material

RA-013-D3RA04259F-s001
