# Supplementary material for: Process of landfill leachate pretreatment using coagulation and hydrodynamic cavitation oxidation
Source: RSC Adv. 2023 Nov 2;13(46):32175–84. doi: 10.1039/d3ra04259f (PMC10620647; doi:10.1039/d3ra04259f)
Supplement: RA-013-D3RA04259F-s001 [file RA-013-D3RA04259F-s001.pdf]

Electronic Supplementary Material (ESI) for RSC Advances.
This journal is © The Royal Society of Chemistry 2023

Supporting Information

Process of landfill leachate pretreatment using coagulation and hydrodynamic cavitation oxidation

Yina Qiao[a,†,*], Riya jin [a,†], Jingshuai Gao [a,*], Kun Wang[a], Jian Xiong[b], MengYe Jia[a], Yu Jiang[a], Zengdi He[a], Jiaoqin Liu[a] ,

[a] *School of Environment and Safety Engineering, North University of China, Shanxi Taiyuan 030051, P.R. China*

[b] *Key Laboratory of Biodiversity and Eco-environmental Protection of the Qinghai-Tibetan Plateau (Ministry of Education), School of Ecology and Environment, Tibet University, Tibet Lhasa 850000, P.R. China*

* Corresponding author.

E-mail address: qiaoyina@nuc.edu.cn (Y. Qiao); 15735055606@163.com (J. Gao).

† These authors contributed equally to this work and should be considered co-first authors.

Total Pages: 3

Texts: 1

Table: 1

Text S1

The calculation of COD value by the following equation:

$$COD_{\mathrm{Cr}} = (V_0 - V_1) \times C \times 8 \times 1000 / V$$

where, C is the ammonium iron(II) sulfate (FAS) standard solution concentration (mol/L); V_0 is the volume of FAS standard solution used when titrating the blank solution (mL); V_1 is the volume of FAS standard solution used when titrating the water sample (mL); V is the water sample volume (mL); 8 is the molar mass of oxygen ($\frac{1}{2}O$) (g/mol).

Table S1. The components and functions of the HC device

device composition	Model and manufacturer	Function
Booster pump	Stainless steel multistage centrifugal pump power: 2.2 kW model: CDLF-100 (Shanghai Yuquan Pump Co., Ltd.).	Provides energy for plant operation, transports wastewater and brings inlet pressure to desired value.
Water tank	PVC, dimensions 500 mm 500 mm 300 mm, thickness: 5 mm, capacity:75 L.	Holds the wastewater solution.
Condensing tube	Rubber hose	Adjust solution temperature to keep temperature constant.
Propeller mixer	Custom built	Solution mixing.
Valves	Stainless steel, DN40 ball valves	Valve 1 is the main valve, which is opened before the booster pump opens. Valves 2,3 and 4 jointly control the inlet and outlet pressure of the HC generator and keep the equipment running constantly.
Pipeline	The main pipeline is made of stainless steel. The side piping is made of PVC and the pipe diameter is DN40.	Constitutes a closed loop, the solution circulates in the cavitation vessel to be degraded and the stainless steel material avoids damage to the pipe due to cavitation in the cavitation process.
Flow meter	Model: LWSY-25, measuring range 1-10 m^3h^{-1} (Dongtai Dongxing Instrument Factory)	Determines the liquid flow of the main line.
Pressure gauge	Model: BD-801 K, Range 0-1.0 Mpa (Shanghai Kaixun Technology Co., Ltd.)	Determines the pressure before and after HC.
